# Modulation of Single-Molecule Emission at Hexagonal Boron Nitride Surfaces

**DOI:** 10.1021/acs.nanolett.5c05814

**Published:** 2026-05-11

**Authors:** Daria Orekhova, Rui Wang, Ze Yu, Jakob Hartmann, Tim Schröder, Niklas Kölbl, Kenji Watanabe, Takashi Taniguchi, Philip Tinnefeld, Sabina Caneva

**Affiliations:** † Department of Precision and Microsystems Engineering, Delft University of Technology, Mekelweg 2, 2628 CD, Delft, The Netherlands; ‡ Department of Chemistry and Center for NanoScience, 9183 Ludwig-Maximilians-Universität München, Munich 80539, Germany; § 52747 National Institute for Materials Science, 1-1 Namiki, Tsukuba, Ibaraki 305-0044 Japan

**Keywords:** hexagonal boron nitride, DNA, fluorophores, photophysics, fluorescence microscopy

## Abstract

Hexagonal boron nitride (hBN) is gaining increasing attention in the field of biomolecule characterization due to its compatibility with single-molecule fluorescence imaging and real-time tracking. Embedding fluorescent molecules within hBN layers offers potential for molecular-resolution sensing devices, since these probes are highly sensitive to their surroundings. Yet, the effect of hBN surfaces on the fluorophore properties remains largely unexplored. Here, we monitor the photophysical properties of ATTO647N-ssDNA on hBN surfaces and elucidate the effects of the environment and substrate. We demonstrate that the presence of hBN increases the photobleaching time and changes intermittency dynamics. By combining van der Waals stacking and FDTD simulations, we subsequently engineer hBN optical cavities to modulate the emission from individual molecules, showing that the brightness can be tuned by a factor of 4. Our findings shed light on light–matter interactions in hybrid nanostructures, which can enable single-molecule imaging and biosensing at high spatial and temporal resolution.

## Graphical abstract

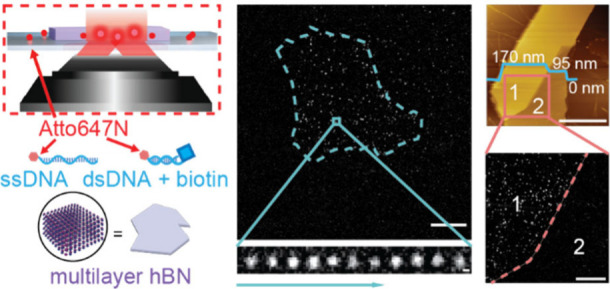

Single-molecule techniques, which probe the sequence, structure, and dynamic behavior of individual molecules, have become crucial components in the biophysical toolbox.^1–5^ Currently, two-dimensional (2D) van der Waals materials, like graphene, transition metal dichalcogenides (TMDs) and MXenes, are finding increasing use in single-molecule sensing devices for biomolecule characterization. The interest in these 2D platforms is driven by their combination of useful chemical, (opto)­electronic, and mechanical properties,^6,7^ which allows engineering of photoexcitations,^8^ dye photodynamics of labeled biomolecules,^9,10^ and label-free sensing schemes.^11^ Van der Waals materials consist of atomically smooth, large-area surfaces that enable adsorption of biomolecules, such as DNA,^9–11^ lipids or amino acids,^12,13^ through a combination of π–π stacking, electrostatic, and hydrophobic interactions.^14–17^ Moreover, 2D materials are particularly attractive due to their high compatibility with a variety of sensing modalities, including nanopores, tunnel junctions, and field-effect transistors.^18–20^

Among 2D materials, hexagonal boron nitride (hBN) is emerging as an alternative platform for single-molecule studies.^21^ It offers increased functionality by enabling direct fluorophore visualization and studies of molecular dynamics relative to conventional substrates such as glass, without requiring specific chemical functionalization for biomolecule attachment.^22,23^ hBN is a wide bandgap (∼6 eV) material that does not exhibit autofluorescence. It is transparent in the visible range and displays an absorption peak in the deep UV range (<210 nm),^24^ thereby precluding fluorescence quenching at pristine hBN surfaces. This property has been harnessed to make atomically precise hBN fluorescence recovery spacers for fluorescent molecules on graphene and metallic substrates.^25,26^ Due to the weakly interacting surface properties, single-molecule fluorescence tracking on hBN revealed fundamental insights into defect-mediated ssDNA diffusion.^27^ The moderate electrostatic force experienced by DNA at hBN surfaces induces adsorption without strongly affecting the dynamical properties (e.g., mobility) of the biomolecules.^11^ These features underlie the potential of hBN surfaces for 1) single-molecule imaging and sensing and 2) as a substrate for real-time investigation of biomolecule motion and interactions. Electronic and ionic transport were also demonstrated across thin hBN flakes,^28–30^ which could pave the way to hBN thickness-dependent sensing of mobile biomolecules across its surface via charge transfer mechanisms.

Bulk exfoliated hBN crystals, however, contain non-emitting defects with an estimated density of around 4 × 10^10^ cm^–2^,^27,31,32^ which can temporarily bind DNA molecules.^11,27^ The spatial arrangement of the defects and their structural properties remain challenging to control. Therefore, the role of crystal defects as nonfluorescent traps and energy transfer sites that can impact photoemission and photodynamics of fluorescent molecules is not yet fully understood.

In this work, we probed fluorophore–hBN photophysics to uncover the impact of hBN on the emission behavior of fluorophores at the single-molecule level. We employed fluorescence imaging in an inverted total internal reflection fluorescence (TIRF) microscope to monitor the photophysical properties of ATTO647N-labeled DNA in different environments and in proximity to hBN surfaces. We analyzed the stability of the fluorescence time traces, brightness, and photobleaching times to gain quantitative insights into hBN–fluorophore coupling.

Our results show that hBN surfaces significantly affect the photophysics of ATTO647N. We found that the introduction of an hBN layer increases the amount of blinking in both air and buffer media. On the other hand, photobleaching times of the fluorophore improved in hBN-containing samples compared with air-exposed samples. Importantly, the photobleaching time for fluorophores under hBN doubled after 90 s of illumination in comparison to fluorophores directly exposed to air. Our results also demonstrate a strong ATTO647N brightness dependence on the hBN thickness, which we ascribe to the formation of an optical cavity defined by the hBN interfaces. Our experimental results are in good agreement with finite difference time domain (FDTD) simulations and show that tuning of the optical cavity length (i.e., hBN thicknesses) can enhance the fluorescence intensity of the hBN-covered regions. The platform ensures planar, noncovalent confinement of single molecules on crystalline surfaces and can function as a testbed for molecular dynamics studies and biomolecular recognition processes with direct optical feedback.

Figure 1a shows the experimental TIRF microscopy setup, where the sample was illuminated from below by a 640 nm laser. The sample consisted of a glass coverslip on which hBN and ATTO647N-labeled DNA were deposited. Fluorescence time traces of the fluorophores were subsequently monitored in five distinct environments, as shown in Figure 1b, namely (1) in buffer with and without Trolox and PCA/PCD complex, (2) in air, and (3, 4, 5) in the presence of hBN (above, below, and encapsulated, respectively). Oxygen scavengers and photostabilizers (PS) were not used during measurements on hBN due to the potential interaction of the micro- to millimolar concentration of proteins contained in these reagents with the hBN flake.^27^ Similarly, the glass coverslip surface was not passivated for measurements with hBN due to the risk of height differences this may introduce and the resulting different near-field interactions. The detailed description of the protocol for each investigated case can be found in Supporting Information S1.2 and 1.3. Figure 1c shows the Raman spectrum of pristine hBN, with the E_2g_ peak at 1365 cm^–1^, which is unchanged when fluorophores are deposited underneath the hBN flake. The extended Raman spectra for the fluorophore/hBN, hBN, and glass references are shown in Supplementary Figure S1. Subsequently, we performed photoluminescence (PL) measurements on the same sample in buffer and found that the peak of ATTO647N at ∼660 nm shifts when measuring the fluorophores under hBN and in air. Specifically, the peak blueshifts by ∼4 nm (under hBN) and by ∼10 nm (in air) (Figure 1d). We note that the Raman and PL spectra originate from emitter ensembles given the laser spot size of ∼1 μm^2^.

**Figure 1:**
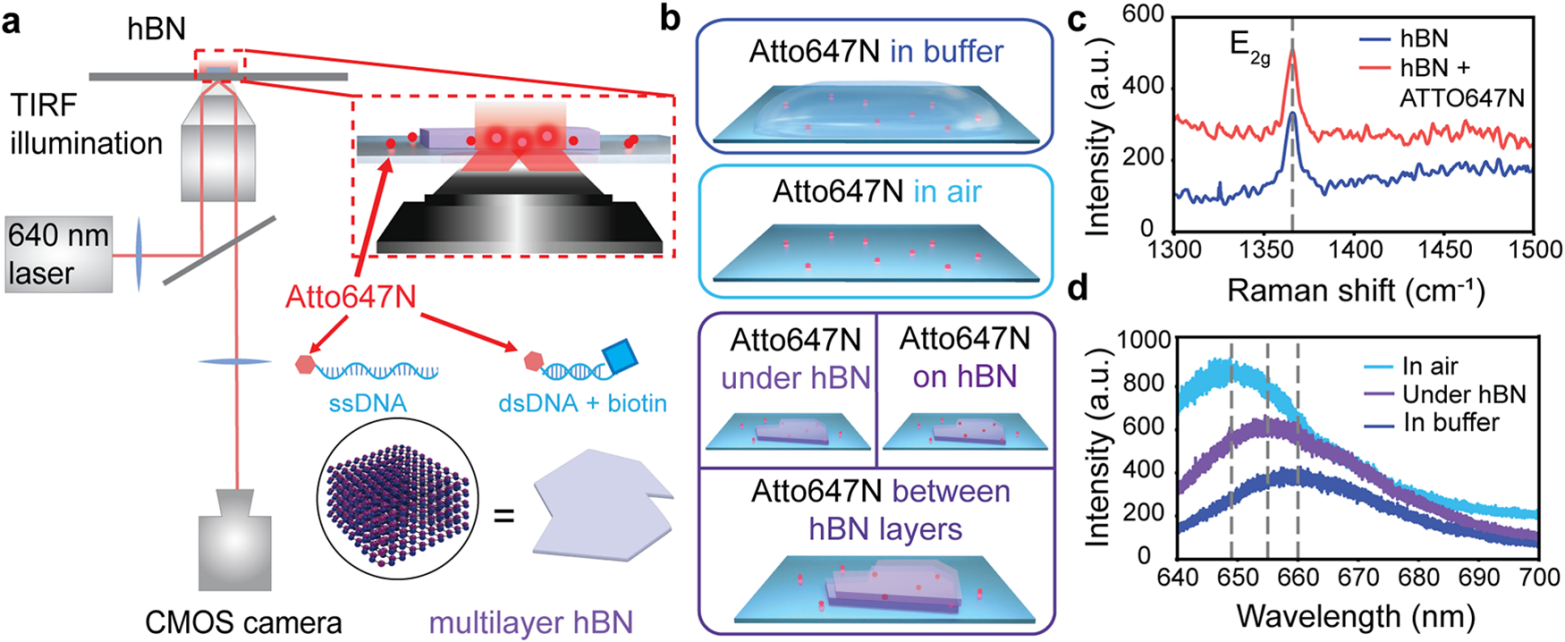
**(a)** Schematic of the TIRF microscopy setup used for single-molecule imaging under 640 nm excitation. The inset illustrates the sample components (ssDNA, biotinylated dsDNA, ATTO647N, hBN). **(b)** The different measured environmental conditions: ATTO647N in buffer with or without photostabilizers (top panel), in air (middle panel), and in/on (in both air and buffer)/between hBN layers (bottom panel). **(c)** Raman spectra of bare hBN and hBN/ATTO647N measured in air. **(d)** PL spectra of ATTO647N in air, in buffer, and under an hBN flake.

The slight blue shift of the PL peak in air with respect to that in buffer was previously observed for various dyes^33,34^ and was attributed to solvatochromic effects.^35^ The red shift under hBN in comparison to that in air can be ascribed to coupling with the substrate, accompanied by the reduction of the HOMO–LUMO gap of the fluorophores and weakening of electron–hole interactions.^36^ Other effects leading to red shifts include screening of the interactions between the transition dipole moments of neighboring molecules^36^ and energy transfer to nonfluorescent traps.^37^

We first investigated the photostability of fluorophores in two environments (buffer, air) and with different substrates (glass, hBN) and extracted time traces during 90 s continuous laser illumination for each case (Figure 2a). In order to characterize the behavior of ATTO647N, we distinguished four different behaviors: stable fluorophores without blinking or bleaching during the measurement period; blinking without bleaching during the measurement time; fluorophore blinking and bleaching; and directly bleaching (Figure 2b). The percentages for each case were calculated from the sum of the molecules across all the flakes in the case (environment of the molecule). The stability of the molecules for each flake in every environment individually, their means, and weighted means can be found in SI Table S2, which takes into account the differences in the number of data points across the flakes. Subsequently, we analyzed the blinking kinetics of the fluorophores by plotting the log–log ‘OFF’ dwell time distribution (Figure 2c).

**Figure 2:**
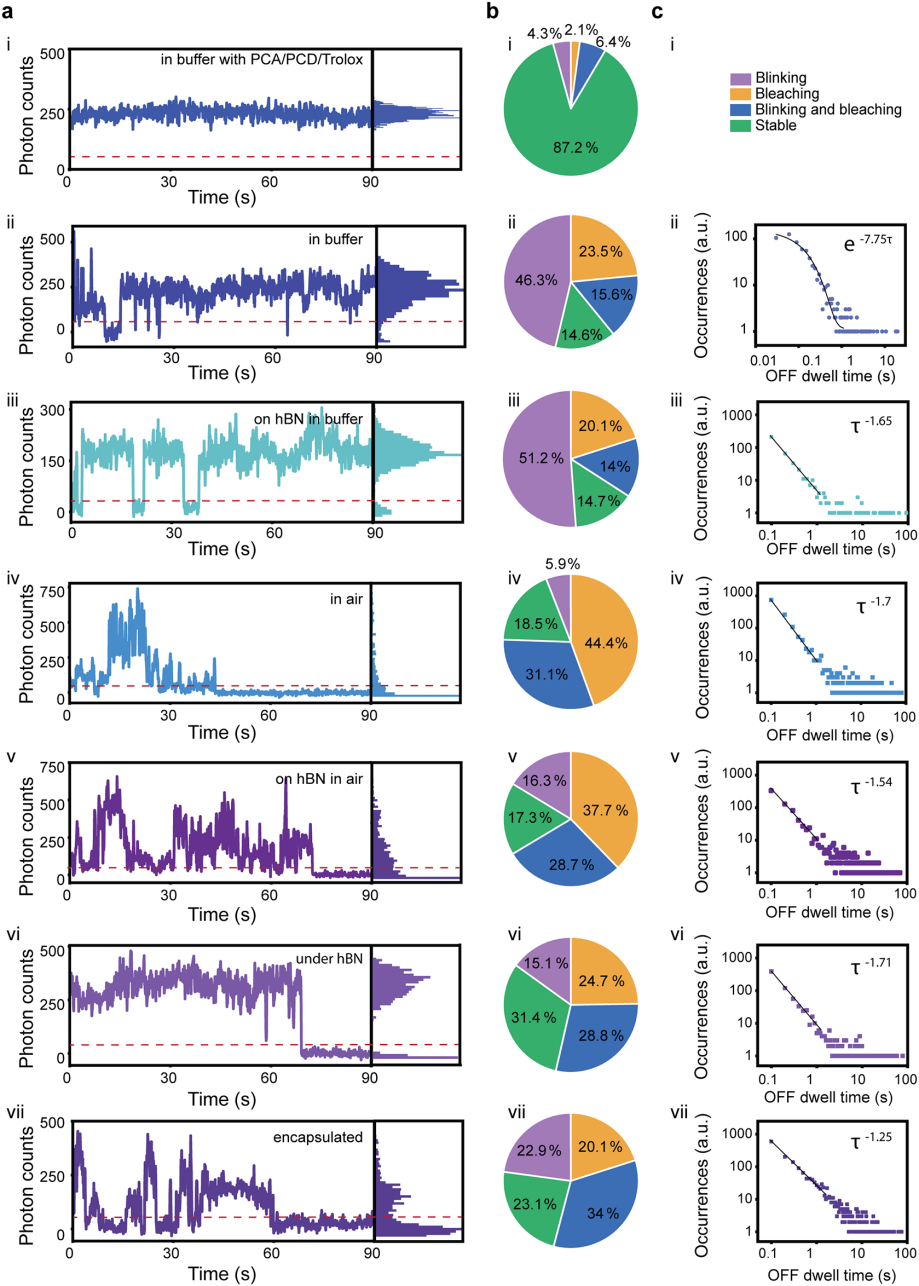
(a) Representative fluorescence time traces and normalized occurrence (right panel) of ATTO647N-DNA i) in buffer with PCA/PCD and Trolox, ii) in buffer without photostabilizers, iii) on hBN in buffer, iv) in air, v) on hBN in air, vi) under hBN, and vii) encapsulated. The red dashed line determines the threshold for defining the ‘ON’ and ‘OFF’ state for each trace presented. (b) Statistics of trace stability for the seven environments respectively during imaging for 90 s. i) *N* = 93 traces, ii) *N* = 473 traces, iii) *N* = 762 traces, iv) *N* = 135 traces, v) *N* = 2837 traces, vi) *N* = 3036 traces, vii) *N* = 503 traces. (c) Log–log “OFF” time distribution for the respective environments.

For the control measurement, ATTO647N-dsDNA in buffer, we used imaging buffer with a reducing agent (Trolox) and oxygen scavengers (PCA/PCD) (Figure 2i). During the measurement, the fluorophore emission remained largely stable during the entire measurement period; that is, ∼87% of the molecules remained in a well-defined ‘ON’ state during continuous laser illumination. We ascribe this robustness against photobleaching to the fast (∼ms) depopulation of the fluorophore triplet state, followed by the complementary redox reaction that brings the fluorophore to the ground state.^38,39^

When oxygen scavengers and photostabilizers were excluded from the buffer (Figure 2ii), we observed many short blinking events (with ∼100–300 ms duration). Overall, 62% of molecules underwent blinking and 40% of the molecules bleached during the experiment, in accordance with previous results in the literature.^40,41^ The intermittency of the ‘OFF’ state of ATTO647N in buffer with molecular oxygen shows a clear single-exponential behavior as expected for a medium containing uniformly distributed electron-acceptor traps of comparable energy levels:^42,43^

$$P\left(\tau_{off}\right)\propto e^{-k_{off}\tau_{off}}$$

where *P*(*τ*_*off*_) is the ‘OFF’ time distribution, *k*_*off*_ is the ‘OFF’ rate constant, and *τ*_*off*_ is the dwell time of the ‘OFF’ state. The single-exponential fit is followed by a heavy tail and distribution broadening at longer time scales.

The degree of intermittency of ATTO647N adsorbed on hBN in buffer (Figure 2iii) showed negligible changes compared to the hBN-free case. However, a change is noticeable in the power law kinetics for the ‘OFF’ dwell times:

$$P\left(\tau_{off}\right)\propto \tau_{off}^{-m_{off}}$$

where *m*_*off*_ is the power law exponent. This behavior can be ascribed to fluorophore–hBN interactions and the existence of multiple nonfluorescent traps with different energies on or near the hBN surface; that is, both the spatial distribution and the energies of the traps are expected to contribute to the variation of charge transfer rates, which lead to the observed wide distribution of dwell times. Probable sources of blinking responsible for different intermittency laws are presented in SI Figure S2. The OFF time distributions observed here with a power law exponent mean of 1.48 (Table S3) are in good agreement with previous work on ATTO647N dyes in aqueous media and embedded in polymer matrices, in which fluorescent blinking was ascribed to dye radical ions formed by photoinduced electron transfer reaction to or from the environment.^42^ Similarly to this work, we find that the OFF time distributions do not inherently obey single-exponential law, but exhibit in one case (ATTO647N in buffer) a monoexponential law dependence, while in the other cases (with hBN and glass substrates) the distributions are best fitted by power law functions. The hBN background fluorescence signal is shown in Figure S3.

In the next step, we investigated whether hBN changes the photophysical properties of the fluorophore in air and whether the photostability of the fluorophore can be improved by the addition of hBN as a dielectric screening layer. ATTO647N was measured in air on the glass coverslip to decouple the effects and contributions of the substrate and the environment. (Figure 2iv). The fluorescence intensity traces showed overall high blinking rates (37% of the fluorophores had at least one blinking event) and short photobleaching times (∼76% of the fluorophores bleached during the measurement). The non-uniform signal, intensity bursts, and rapid bleaching can be attributed to the increased concentration of molecular oxygen in the surroundings, depopulating the triplet state.^38^ As ATTO647N was measured in dry conditions, the restriction of the rotational freedom of the dye could have led to the enhanced brightness and quantum yield, explaining intensity differences with respect to a fluorophore in buffer media.^44–46^ The intensity fluctuations, on the other hand, can be attributed to charge tunneling from the excited state of the molecule to charge traps in the glass.^35,47,48^ The results obtained for the fluorophores in air agree with observations from literature^35,47,48^ and obey the power-law behavior for the “OFF” state.

Next, we monitored ATTO647N traces on hBN in air (Figure 2v). The photostability of the fluorophores did not significantly improve, with ∼17% of traces measured for various hBN thicknesses representing fully stable molecules, a similar percentage to those directly on glass. We ascribe bleaching effects to the detrimental effect of molecular oxygen,^38,42^ while blinking may be due to the intermolecular charge transfer between the excited state of the fluorophore and hBN traps. This could be analogous to previous work where blinking of ATTO647N embedded in polymer matrices was ascribed to charge transfer between the dye and the localized states found in the polymer matrix. The number of blinking molecules increased by 8%.

Finally, in order to remove oxygen from the environment, we investigated ATTO647N under hBN and encapsulated it between hBN layers (Figure 2vi,vii). ATTO647N under hBN was protected from molecular oxygen, which led to more stable traces during the measurement time: 34% of the molecules remained stable, almost twice the amount of the fluorophores in air (Figure 2iv). Interestingly, in these experiments, we also noticed that 44% of the molecules were blinking, which can be explained by the removal of molecular oxygen from the system.^38^

By encapsulating the fluorophore between the hBN layers, we hypothesized achieving full or partial blinking suppression, an effect previously observed for passivated single molecules or quantum dots in trap-free polymer matrices.^49,50^ Moreover, encapsulation of solid-state hBN emitters by addition of top and bottom hBN crystal layers was previously used to stabilize these optically active defects.^51^ However, while the encapsulation of molecules between hBN flakes led to longer bleaching lifetimes compared to one-sided hBN encapsulated ones, it did not lead to enhanced stability, with 57% of the molecules blinking during the measurement time, challenging the notion that hBN is fully inert (Figure 2vii). This increased percent of blinking in comparison to the encapsulation between the hBN/glass interface suggests the presence of fluorophore–hBN defect interactions, which becomes more pronounced with two hBN interfaces.

Charged defects with various structures are known to exist in pristine hBN^52^ and can interact with organic solvent molecules, with the latter changing their dynamics and photophysics as they hop or diffuse between defect-binding sites. Charge-donating or -accepting behavior was previously also reported for single terrylene molecules interacting with hBN lattice defects, changing the fluorescence emission spectrum of these molecules.^53,54^ Such defects may be responsible for the enhanced blinking observed here, especially for fully encapsulated fluorophores. The behavior of ATTO647N coupled to hBN is more complex and cannot be fitted with a single-exponential power law (Figure 2c).

Our experiments suggest that by shielding fluorophores from interactions with oxygen and glass, full and partial hBN encapsulation improves the bleaching lifetime of the molecules. Figure 3a further details this effect, illustrating an hBN flake with ATTO647N deposited on top of an hBN flake in buffer, delineated by the blue dotted line. The insets on the bottom are temporal snapshots of a single representative molecule over time for a duration of 90 s. The comparison of the photobleaching time in all tested environments, measured as the percentage of fluorophores remaining emissive after 90 s exposure, is shown in Figure 3b, where samples imaged in the presence of photostabilizers are accompanied by the abbreviation PS. Overall, the introduction of hBN for both liquid and dry measurements helps to increase the photobleaching times. While in buffer this increase is not as prominent, dry measurements of ATTO647N on the hBN surface show an increase in the photobleaching times by on average ∼35% in comparison to air. Measurements of molecules under or encapsulated between hBN flakes show 2–2.5 times increase in the photobleaching times in comparison to direct exposure to air.

**Figure 3:**
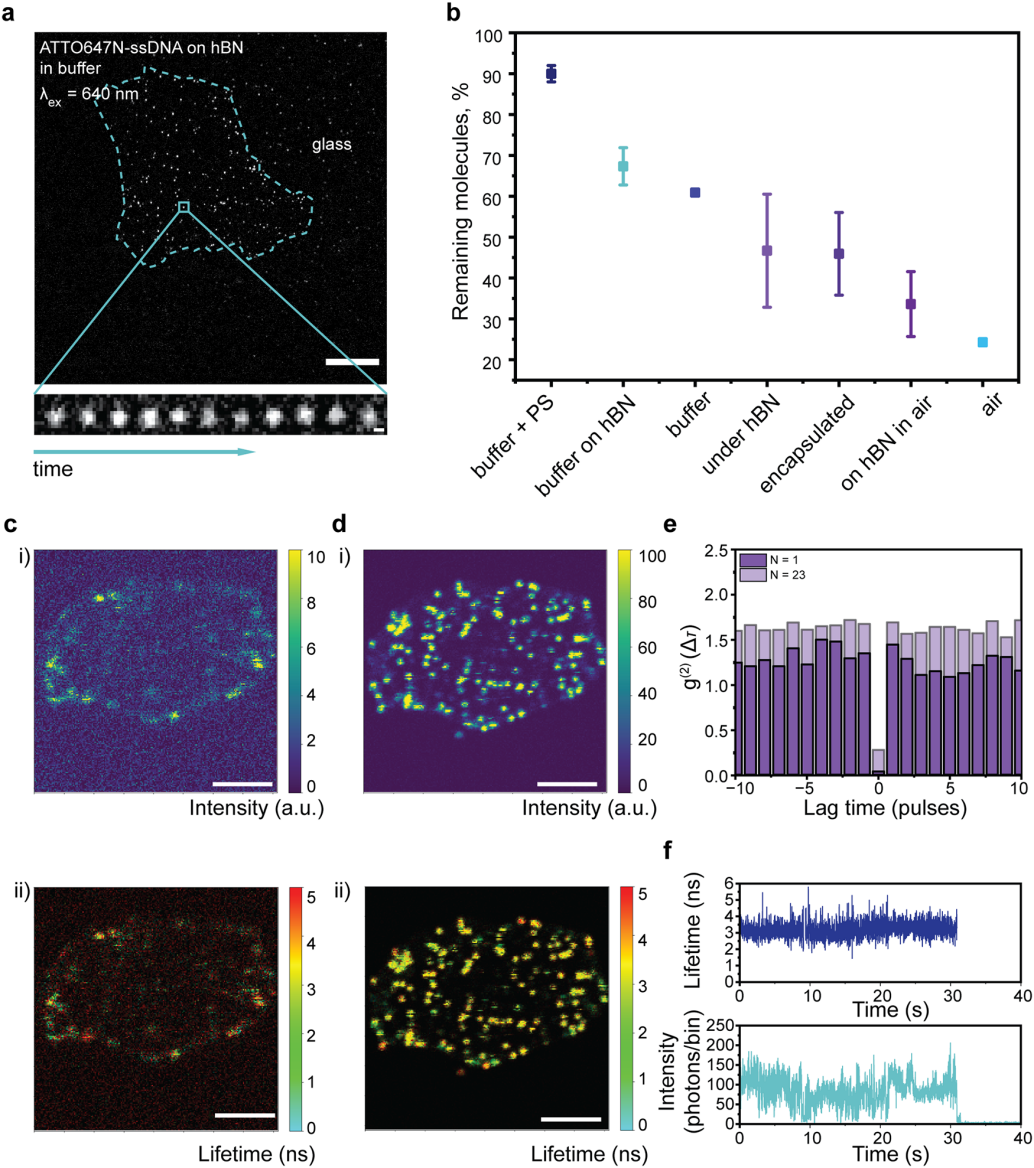
(a) hBN with ATTO647N on top in buffer. The blue dotted lines represent the outline of the hBN flake. Scale bar is 10 μm. The panel on the bottom shows the blinking of a representative fluorophore during 90 s illumination. Scale bar is 2 μm. (b) Percent of remaining molecules after 90 s continuous laser illumination under different conditions. The abbreviation “PS” stands for photostabilizers in the buffer. (c) Fluorescence intensity (i) and FLIM (ii) of bare hBN in 10 mM Tris/1 mM MgCl_2_ buffer. (d) Fluorescence intensity (i) and FLIM (ii) of typical ATTO647N-ssDNA deposited on hBN in buffer. Scale bar for all the images is 5 μm. (e) Extracted second-order correlation function for one molecule and the weighted mean of 23 molecules. (f) Typical lifetime and intensity change over time of ATTO647N deposited on top of hBN plotted with 10 ms binning. A threshold of 20 photon counts was used for calculating the lifetime change over time.

We have additionally performed correlative emission intensity and FLIM (fluorescence-lifetime imaging microscopy) measurements of fluorophores on top of hBN, where the set of 27 molecules was measured on top of 1 flake. The hBN flake in buffer before fluorophore deposition shows a low emission background (Figure 3c). Upon deposition of ATTO647N-ssDNA on top of the same flake, the intensity increases by approximately a factor of 10, allowing clear localization of the molecules (Figure 3d). Measurements of the second-order correlation function, in which characteristic photon antibunching^55^ was observed, with *g* ^(2)^(0) < 0.3, prove the single-molecule nature of the emission spots. Data for a typical molecule (dark purple) and an average of 23 molecules (light purple) are shown in Figure 3e. As we have chosen the spots stochastically, we have sometimes also observed multiple emitters with distinct bleaching steps (4 traces in total). The corresponding *g* ^(2)^(0) change for the relevant segments of one of these traces can be found in Supplementary Figure S4. The correlation function for single emission spots corresponding to multiple emitters was not included in the analysis in Figure 3e. Moreover, we concurrently measured the lifetime and intensity over time for one of the fluorophores (Figure 3f). The lifetime signal remains stable at ∼3.5 ns and is largely independent of the intensity fluctuations. This behavior appears to indicate that a static quenching mechanism is at the basis of the interaction between hBN and the fluorophore, thus the formation of the nonemissive ground state complex.^56^ From a set of 27 fluorophores measured on top of one flake, 24 showed this behavior. Conversely, the remaining 3 molecules showed a dynamic quenching mechanism, where the lifetime and intensity synchronously changed over time. For these molecules, the lifetime and *g* ^(2)^(0) are presented in Supplementary Figure S5.

Finally, we investigated the effect of hBN thickness on the fluorophore photobleaching time and brightness. The photobleaching rate, plotted as the normalized number of molecules as a function of time, for a range of thicknesses of 10–250 nm can be found in Figure S6, showing that there is no linear dependence of photobleaching time with hBN thickness. In the cases of Figure S6­(c–f), we attribute the improved temporal stability in the presence of hBN to protection from molecular oxygen combined with a lack of redox agents to escape the triplet state.^38^

Strikingly, we observed that fluorophore intensities are strongly hBN thickness-dependent in the 10–250 nm thickness range and that fluorescence persisted under relatively thick hBN flakes (>150 nm). Figure 4a shows the optical image of two representative terraced hBN flakes with ATTO647N underneath. Figure 4b,c shows AFM images and corresponding fluorescence images of ATTO647N. From the TIRF images, the fluorescence intensity drastically changed between 95 and 170 nm layers in the first flake (regions 1 and 2, respectively), with fluorophores being much dimmer under 95 nm hBN. Conversely, thinner layers with relatively smaller height changes (30 and 23 nm, regions 3 and 4, respectively) on the second flake had a minimal effect on the brightness of the fluorophores.

**Figure 4:**
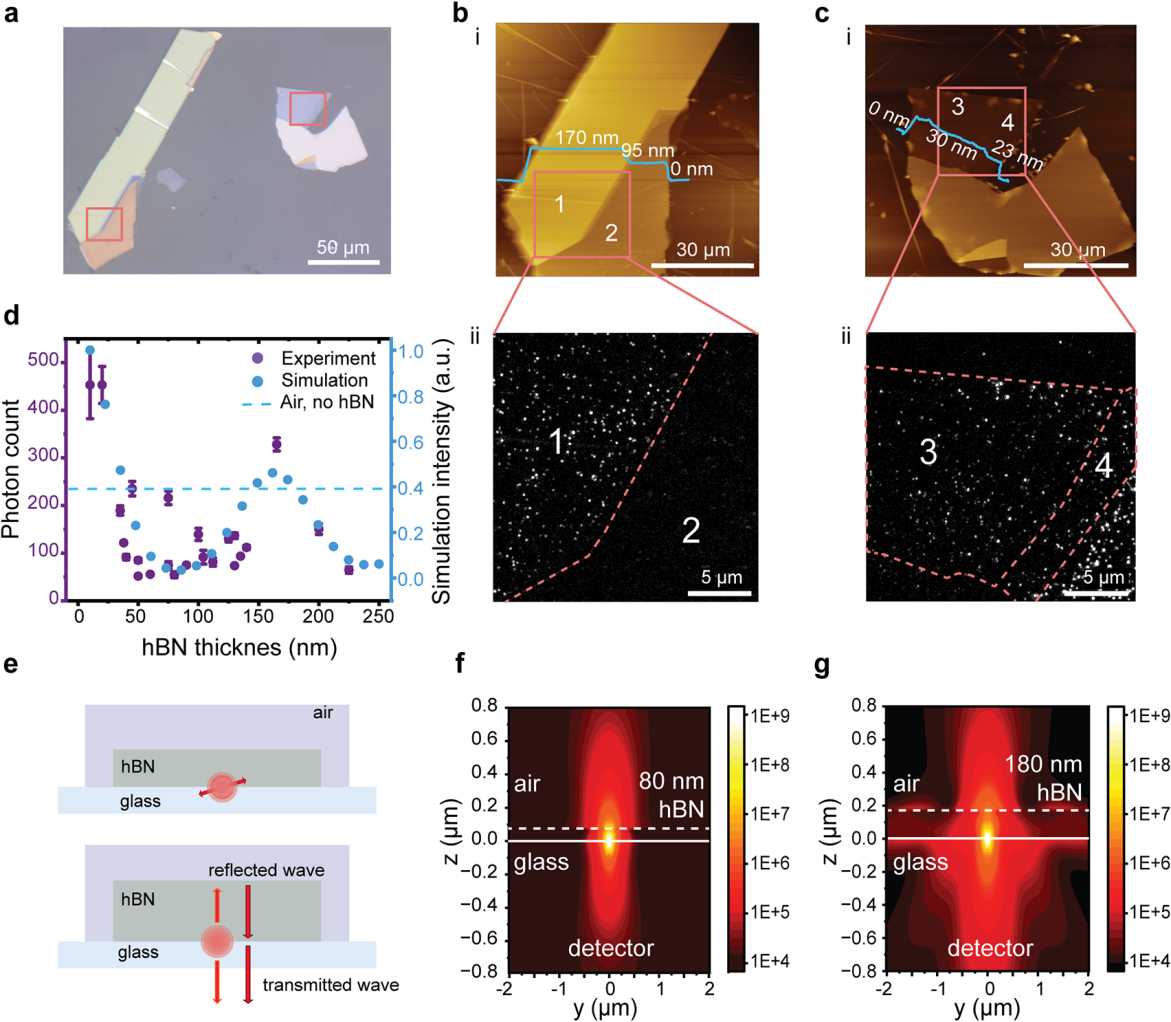
(a) Optical image of two measured hBN flakes on a glass substrate. (b, c) Corresponding AFM images of the measured regions (i) and fluorescence signal from ATTO647N-ssDNA underneath the flakes (ii). (d) Experimental and simulated results of fluorophore brightness variations per frame under hBN flakes of different thicknesses. (e) Polarization of the dipole (top panel) and thin film interference (bottom panel) influencing the emission intensity. (f, g) Simulated electric field of a dipole source under 80 and 180 nm thick hBN. The white line shows the glass/hBN interface, located at *z* = 0. The white dashed line shows the hBN/air interface.

We analyzed the effect for hBN flakes in the 10–250 nm thickness range and compared it with finite-difference time-domain (FDTD) simulations, in which the emission intensity (brightness) was monitored across the same hBN thickness range.

From Figure 4d, the highest brightness is achieved when using either thin flakes (10–20 nm) or relatively thick ones (∼160–170 nm). In these regions the intensity of the fluorophore can reach up to 500 photon counts per frame after background correction (i.e., 5000 photons/s). These peaks are surrounded by low brightness valleys (50–120 nm and 190–250 nm hBN thickness), where intensity drops down to 50 counts per frame (i.e., 500 photons/s). The oscillating behavior of the brightness excludes a simple linear thickness effect of hBN. The intensity of ATTO647N directly deposited on glass and exposed to air, i.e., corresponding to 0 nm hBN thickness, is also included in the plot as a horizontal dashed line and shows a lower intensity than with thin (<25 nm) hBN coverage. The photon count after background correction corresponds to ∼200–250 counts per frame.

In the FDTD simulations, fluorophores were modeled as dipole sources at the interface of hBN and glass (schematically represented in Figure 4e). Since the incident light is linearly polarized, the dipole source is in-plane oriented due to the photoselection process of dipole polarization (see Figure S7).^57^ The details of the simulation are provided in the SI (S1.6). To investigate the brightness enhancement, the simulation considered three processes: the excitation enhancement, quantum efficiency enhancement, and collection efficiency enhancement. The response of each of these effects individually can be found in Figure S8. In Figure 4d, the total simulated brightness is strongly modulated by the hBN thickness, in good agreement with the experimentally measured values. The outliers in the plot can be attributed to random ATTO647N deposition onto the glass substrate before hBN placement, allowing for the presence of double fluorophores in the same spot, as well as fluorophore–hBN trap coupling. Notably, brightness minimum and maximum values are obtained at hBN thicknesses of 80 and 180 nm, respectively, which correspond to a maximum enhancement factor of 4-fold. The simulated electric field distributions with these two hBN thicknesses, shown in Figure 4f,g, respectively, are in agreement with the imaging results.

Thus, selection of the hBN thickness provides a tuning knob to modulate the intensity of the fluorophores, leading to either enhanced or reduced brightness. These results could be explained by the presence of a Fabry–Pérot cavity defined by the bottom hBN and top hBN interfaces, where the cavity length (i.e., here the hBN thickness) determines the regimes for constructive and destructive interference (see SI1.6 and Figure S9).^58^ Similar results were obtained for the ATTO647N on top of hBN in air (see Figure S10).

Finally, we note that the results presented here for ATTO647N are not universal for different dyes. Dyes of different charge show different intermittency dynamics, which suggests different interactions with hBN. ATTO647N has a charge of +1, which can lead to the dye accepting electrons from negatively charged hBN defects (such as V^–^_B_),^59^ thereby affecting intermittency dynamics. As a comparison, we subsequently tested ATTO647, which has a neutral charge, in air on top of hBN, while keeping the same ssDNA length and sequence, and extracted a stretched exponential OFF time distribution (Figure S11).

In summary, we investigated the influence of 2D hBN on the photophysics of ATTO647N-ssDNA while varying the medium conditions and substrates. We performed single-molecule imaging for five different cases: ATTO647N in buffer and air and in proximity to hBN surfaces (above, below, and encapsulated). Introducing hBN on top and/or underneath fluorophores led to an increased blinking rate, which was especially prominent during hBN encapsulation. Given the presence of inherent crystal defects in pristine hBN, these dynamics can be attributed to fluorophore–hBN defect interactions. hBN top coverage is found to stabilize fluorophores in air, leading to an almost 2.5 times longer bleaching lifetime after 90 s of continuous exposure. Importantly for experiments in physiological conditions, we find that the majority of fluorophores on hBN remain in an active (fluorescent) state (∼66%), while still blinking. We also demonstrate the generation of an optical cavity, where rational selection of hBN flake thickness allows tuning of the fluorophore brightness. This enables the deterministic design of vertical material stacks for optimized fluorescence intensity measurements.

By integrating fluorophores with pristine hBN, our results show high sensitivity of the fluorophores to changes in their optical near-field. Interfacing organic emitters and inorganic matrixes at the nanoscale can thus be harnessed to engineer single-molecule photophysics and expand hBN-based frameworks for single-molecule sensing and imaging. The ability to precisely control the distance between fluorophores underneath and above hBN, which is tunable with single-atomic-layer resolution, can form the basis for both energy-transfer and electron-transfer detection schemes. Beyond applications in optical nanoscopy, we foresee that with the use of dispersive elements in the emission pathway the platform can be extended to spectroscopic analysis, providing multiparameter characterization. Finally, deterministic placement of fluorophores via precise positioning on DNA origami nanostructures can lead to massively parallel single-molecule sensing, avoiding self-quenching from closely spaced fluorophores.^60^ This planar, on-chip imaging modality can thus serve in next-generation bio-nanophotonic devices for both fundamental studies at the single-molecule level and applications in molecular diagnostics.

## Supplementary Material



## Notes

Materials and Methods used in this work: hBN flake sample preparation; DNA sample preparation; TIRF sample preparation; ATTO647N/hBN characterization; fluorescence intensity trace analysis and OFF dwell times distribution; FDTD simulation; supporting figures: PL of ATTO647N under hBN and Raman peak of hBN on glass; second-order correlation function measurement of ATTO647N; Jablonski diagram describing the formation of dark states for the fluorophore on different substrates and media; second-order correlation function for multiple emitters; dynamic quenching of ATTO647N; normalized number of molecules as a function of time on and under hBN of different thickness; comparison of the fluorophore intensity with Ey and Ez polarization, simulated for fluorophores underneath the hBN surface; the effect of the excitation enhancement, quantum efficiency enhancement and collection efficiency enhancement on the fluorophore brightness under hBN; thin-film interference in the system with hBN serving as a Fabry–Pérot cavity; brightness modulation of ATTO647N measured on top of hBN in air; ATTO647 OFF time distribution (PDF)

## ¶

State Key Laboratory of Medicinal Chemical Biology, Tianjin Key Laboratory of Molecular Recognition and Biosensing, Frontiers Science Center for New Organic Matter, College of Chemistry, Nankai University, Tianjin 300071, China
