# Supplementary material for: Modulation of Single-Molecule Emission at Hexagonal Boron Nitride Surfaces
Source: Nano Lett. 2026 May 11;26(19):6260–9. doi: 10.1021/acs.nanolett.5c05814 (PMC13195644; doi:10.1021/acs.nanolett.5c05814)
Supplement: Supplementary file 1 [file nl5c05814_si_001.pdf]

Supporting Information

Modulation of Single-Molecule Emission at Hexagonal Boron Nitride Surfaces

Daria Orekhova[1], Rui Wang[1], Ze Yu[1], Jakob Hartmann[2], Tim Schröder[2], Niklas Kölbl[2], Kenji Watanabe[3], Takashi Taniguchi[3], Philip Tinnefeld[2], Sabina Caneva[1]*

[1] Department of Precision and Microsystems Engineering, Delft University of Technology, Mekelweg 2, 2628 CD, Delft, The Netherlands

[2] Department of Chemistry and Center for NanoScience, Ludwig-Maximilians-Universität München, Munich 80539, Germany

[3] National Institute for Materials Science, 1-1 Namiki, Tsukuba, Ibaraki 305-0044 Japan

*E-mail: s.caneva@tudelft.nl

Table of content

1. **Materials and Methods**
    - **1.1 hBN flake preparation**
    - **1.2 DNA sample preparation**
    - **1.3 TIRF sample preparation**
    - **1.4 ATTO647N/hBN characterization**
    - **1.5 Fluorescence intensity trace analysis and OFF dwell times distribution**
    - **1.6 FDTD simulations**
2. **Supporting figures**
    - **2.1 Figure S1. PL of ATTO647N under hBN and Raman peak of hBN on glass**
    - **2.2 Figure S2. Second-order correlation function measurement of ATTO647N**
    - **2.3 Figure S3. Jablonski diagram describing the formation of dark states for the fluorophore on different substrates and media**

2.4 Figure S4. Second-order correlation function for multiple emitters
2.5 Figure S5.Dynamic quenching of ATTO647N
2.6 Figure S6. Normalized number of molecules as a function of time on and under hBN of different thickness
2.7 Figure S7. Comparison of the fluorophore intensity with E_y and E_z polarization, simulated for fluorophores underneath the hBN surface
2.8 Figure S8. The effect of the excitation enhancement, quantum efficiency enhancement and collection efficiency enhancement on the fluorophore brightness under hBN
2.9 Figure S9. Thin-film interference in the system with hBN serving as Fabry-Perot cavity
2.10 Figure S10. Brightness modulation of ATTO647N measured on top of hBN in air
2.11 Figure S11. ATTO647 OFF time distribution

References

1. Materials and Methods

1.1. hBN flake preparation

hBN bulk crystals were gifted by the National Institute for Material Science (Japan). hBN nanosheets were prepared via exfoliation with the sticky tape (Magic Scotch tape, 3M) and then transferred to the cleaned glass substrate. No other treatment of hBN was implemented.

1.2. DNA sample preparation

Single-stranded DNA (ssDNA) strands were purchased from Merck (Sigma Aldrich) and were used without further purification. For each measurement, the ssDNA was diluted to the desired concentration in 1xTAE, 10 mM $MgCl_2$ buffer. First, the following sequence (Strand 1) with ATTO647N label was used: 5'-ATTO647N-CGGCAAAATCCCTTTAGGGGTGGTTCCGA-3' (29 nt). In order to perform the neutravidin-biotin linkage, the second strand partially complementary to this one was used: 5'-biotin-AAAGGGATTTTGCCGAATGATAGACGGTTTTT-3' (Strand 2). The same two sequences with Strand 2 without biotin (Strand 3) were used for creating ssDNA-dsDNA-ssDNA (14-15-17 bp respectively) construct for deposition of DNA on top of hBN in buffer, thus anchoring the molecules in one place during the measurement.

1.3. TIRF sample preparation

For the measurements in air, glass coverslips of thickness 1.5H were initially cleaned using ultrasonication in acetone, followed by IPA and Milli-Q water. The treated slips were dried using air gun and oxygen plasma cleaned for 10 min (0.16 Mbar, 80 mW).

For measurements under hBN, 10 nM of ssDNA modified with ATTO647N was put on the coverslip and incubated for 1 minute. In order to avoid drying and aggregation, the remaining liquid was immediately washed away using an air gun. Coverslips were heated on the hotplate for

3-5 min at 150˚C in order to evaporate the remaining liquid and fix the molecules at one place. Afterwards the hBN flakes were exfoliated onto the glass substrate as described in the above paragraph.

For measurements on top of hBN in air and encapsulated between hBN layers, 5-10 pM ssDNA in 1xTAE, 10mM $MgCl_2$ buffer was deposited into the surface and then dried using the gentle airgun flow. For encapsulation measurements, the top hBN flake was transferred via a PDMS dome with PPC film.

For measurements on top of hBN flake in buffer, the hBN flake was directly exfoliated to the cleaned glass coverslip. Afterwards the droplet of ssDNA-dsDNA-ssDNA in 1xTAE, 10mM $MgCl_2$ was added on top of the flake. This construct allowed to limit the lateral movement of the fluorophore without using any chemical linker.

For the measurements in buffer without hBN, the glass chambers were first made and cleaned with KOH. Afterwards dsDNA modified with ATTO647N was immobilized to the surface via a biotin-neutravidin linker. Imaging buffer, containing 1xTAE, 10mM $MgCl_2$. Trolox and PCA/PCD was added for photostabilization. The chamber was sealed to prevent interactions with molecular oxygen.

All the samples were prepared just before the measurement and had the similar degree of the pre-measurement light exposure.

Table S1. Comparison table for all the cases investigated

Media	Media specification	Was hBN present?	DNA used
buffer	1xTAE, 10mM $MgCl_2$	Yes, with fluorophores above	Strand 1+ Strand 3
	1xTAE, 10mM $MgCl_2$	No	Strand 1+ Strand 2
	1xTAE, 10mM $MgCl_2$, Trolox, PCA, PCD	No	Strand 1+ Strand 2
air	-	no	Strand 1
	-	Yes, with fluorophores below	Strand 1
	-	Yes, with fluorophores above	Strand 1
	-	Yes, with fluorophores encapsulated	Strand 1

1.4. ATTO647N/hBN characterization

Fluorescence traces were obtained with Nikon inverted microscope (Nikon Instruments, Eclipse Ti2) with a 100x oil objective (Nikon Instruments, Apo SR TIRF ×100, NA1.49). TIRF illumination was used for better contrast. All the samples excluding the ATTO647N in buffer dwell time distribution (Figure 2c-ii) were illuminated with 640 nm fibre laser with total of 2 mW laser power and 100 ms exposure time.[1-3] To obtain the OFF time duration statistics for the ATTO647N in buffer without photostabilizers (Figure 2c-ii), the sample as illuminated with 640 nm fibre laser with total of 12 mW and 30 ms exposure time. A bandpass filter (661-690) was used in the emission pathway. Images were collected with a sCMOS camera (Prime BSI Express, Teledyne) with an effective pixel size of 43 nm. Typical photon counts rate after background correction contained up to ~20 counts for the background and ~200-250 counts for the ATTO647N on glass. Such SNR was chosen to get the clear transition between the ON/OFF state and clearly distinguish the blinking events.

AFM measurements of the hBN flake thicknesses were performed on a JPK Nanowizard 4 using static force mode. CONTR-50 tips were used. The background correction and height extraction were made in Gwyddion.

Photoluminescence spectra of the flakes and ATTO647N were measured with Horiba Raman Spectroscope under 632 nm excitation and 5 s acquisition time.

Second order correlation function measurements and lifetime measurements (Figure S2) were pursued at Luminosa Single Photon Counting Microscope (PicoQuant GmbH). For this the ATTO647N-ssDNA was deposited on top of hBN flake. The probe of bare hBN flake in buffer was always measured beforehand to determine the background. The probe was excited via 640 nm pulsed laser with 0.8 μW power and 25 MHz repetition rate. Signal was separated by 50/50 beam splitter and registered using 2 separate SPAD detectors.

The correlation function and lifetime change were then extracted and plotted using the home-written python script. For extraction of the second-order correlation function for each trace the microtime gating was used.[4] In this way we increased SNR and got rid of the laser scattering. The mean second order correlation function across the traces was calculated as the weighted mean of the correlation function for single molecules.

Lifetime over time change was extracted via the MLE tail fit and took the background into account. The threshold of 20 counts was taken as the minimum trace intensity for the lifetime extraction.

1.5. Fluorescence intensity trace analysis and OFF dwell times distribution

Data analysis of the fluorescence traces was made via iSMS[5] and FijiJ TrackMate plugin. Beforehand the background correction via rolling ball as well as drift correction were implemented for all videos. For traces obtained from iSMS at first the home-written script performing Hidden

Markov Model analysis was used to determine blinking and bleaching events. Afterwards, the thresholding was implemented for each molecule separately due to HMM imperfection and hBN possible background signal. For the OFF dwell times only the blinking events reaching the background photon count were considered, which could imply the single-molecule nature of blinking events. Fitting of OFF time constants was made via Origin data processing.

Detailed percentage of stable, blinking, blinking& bleaching and bleaching fluorophores for each flake can be found in the table S2 below. The means and weighted means for each case were calculated. Weighting was made according to the number of traces extracted from each video.

Table S2. Traces stability for all the investigated hBN flakes

**Case**	**hBN thickness**	**Stable (%)**	**Blinking (%)**	**Blinking& Bleaching (%)**	**Bleaching (%)**
Under hBN	10 nm	0	0	18.18	81.82
	20 nm	8.89	2.22	22.22	66.67
	35 nm	59.52	10.71	14.29	15.48
	38 nm	47.11	15	24.21	13.68
	40 nm	55.36	14.29	12.5	17.86
	45 nm	6.15	3.08	36.92	53.85
	50 nm	45.25	14.53	28.49	11.63
	60 nm	19.08	22.37	36.18	22.37
	75 nm	35.6	17.96	35.29	11.15
	90 nm	32.35	14.71	35.29	17.65
	100 nm	16.49	15.46	31.96	36.08
	104 nm	17.39	26.09	34.78	21.74
	112 nm	7.89	17.11	34.21	40.79
	125 nm	25.93	7.41	37.04	29.63
	130 nm	40.48	9.52	33.33	16.67
	130 nm	30	6.67	30	33.33
	135 nm	23.58	15.09	17.92	43.4
	140 nm	27.81	18.93	34.91	18.34
	165 nm	31.05	14.16	29.91	24.89
	200 nm	22.22	15.28	26.39	36.11
	225 nm	34.02	16.49	20.62	28.87
	Mean+SD	27.91±15.58	13.19±6.35	28.32±7.71	30.57±18.14
	**Weighted mean + SD**	**32.45±12.56**	**15.18±4.02**	**28.56±6.46**	**23.8±12.54**
	20 nm	20.27	9.46	22.97	47.3

Above hBN in air	21 nm	20.75	24.53	22.64	32.08
	28 nm	29.89	26.44	21.84	21.84
	30 nm	8.74	10.68	36.89	43.69
	33 nm	17.17	24.24	23.74	34.85
	35 nm	11.02	16.54	33.86	38.58
	60 nm	13.94	12.02	28.85	45.19
	85 nm	25.88	16.08	27.06	30.98
	95 nm	22.73	13.64	29.55	34.09
	120 nm	15.79	20.65	26.72	36.84
	130 nm	10.41	14.93	37.1	37.56
	140 nm	18.18	19.16	28.57	34.09
	150 nm	19.17	17.5	30.83	32.5
	150nm	19.75	15.43	24.07	40.74
	225 nm	12.99	17.53	33.12	36.36
	265 nm	20	16.84	35.79	27.37
	650 nm	16.8	9.45	25.98	47.77
	Mean+SD	17.85±5.35	16.77±4.93	28.8±4.95	36.58±6.74
	**Weighted mean + SD**	**17.27±4.83**	**16.32±4.67**	**28.69±4.42**	**37.72±6.44**
Above hBN in buffer	-	32.38	33.33	4.76	29.53
	-	13.62	52.49	10.63	23.36
	-	10.39	55.34	19.66	14.61
	Mean+SD	18.8±9.69	47.05±9.77	11.68±6.13	22.5±6.12
	**Weighted mean + SD**	**14.7±7.22**	**51.18±7.25**	**14.04±5.58**	**20.12±5.52**
Encapsulated between hBN flakes	95 nm	18.37	26.53	33.67	21.43
	105 nm	12.88	23.31	44.79	19.02
	130 nm	35.29	16.99	25.49	22.22
	150 nm	33.33	28.33	21.67	16.67
	240 nm	10.34	27.59	44.83	17.24
	Mean+SD	22.04±10.37	24.55±4.15	34.09±9.57	19.31±2.2
	**Weighted mean + SD**	**23.05±10.26**	**22.86±4.25**	**34±9.2**	**20.08±1.99**

The detailed OFF times constants for all the hBN thicknesses including the standard deviations (SDs) are presented in the Table 3. OFF rate constants were not obtained for some flakes due to the low amount of blinking events detected for reliable statistics.

Table S3. OFF rate constants with standard deviations (SD) for all the investigated cases as well as the number of molecules (traces) for each case

**Case**	**hBN thickness**	**Number of traces**	**Law**	**OFF rate constant**	**SD**
Buffer without PS	-	473	Single exponential law	11.24	0.63
Air	-	135	Power law		
Under hBN	10 nm	11	Power law	0.73227	0.05486
	20 nm	45	Power law	0.99641	0.02327
	35 nm	84	Power law	1.83308	0.04138
	38 nm	380	Power law	1.71023	0.01522
	40 nm	56	-	-	-
	45 nm	65	Power law	1.77797	0.09016
	50 nm	172	Power law	-	-
	60 nm	152	-	-	-
	75 nm	323	Power law	1.29368	0.02501
	90 nm	34	-	-	-
	100 nm	97	Power law	0.84716	0.09201
	104 nm	23	Power law	1.05189	0.06641
	112 nm	76	-	-	-
	125 nm	27	Power law	2.38988	0.13102
	130 nm	42	-	-	-
	130 nm	30	Power law	2.40235	0.07503
	135 nm	106	Power law	1.15687	0.05889
	140 nm	169	Power law	1.98472	0.20185
	165 nm	438	Power law	1.38443	0.03625
	200 nm	144	Power law	2.05584	0.10295
	225 nm	194	-	-	-
Above hBN in air	20 nm	74	Power law	1.20682	0.02978
	21 nm	53	Power law	1.19876	0.02808
	28 nm	87	Power law	1.30688	0.01744
	30 nm	103	Power law	1.4702	0.02277
	33 nm	198	Power law	1.38182	0.01786
	35 nm	127	Power law	1.11509	0.02218
	60 nm	208	Power law	1.26603	0.0176
	85 nm	255	Power law	1.64768	0.03587
	95 nm	44	Power law	1.70237	0.05739
	120 nm	247	Power law	1.6203	0.02207
	130 nm	221	Power law	1.26888	0.02357
	140 nm	308	Power law	1.33481	0.0112

	150 nm	120	Power law	1.54253	0.04446
	150nm	162	Power law	1.50935	0.02907
	225 nm	154	Power law	1.57965	0.00642
	265 nm	95	Power law	1.3153	0.05472
	650 nm	381	Power law	1.39769	0.02011
Above hBN in buffer	-	105	Power law	1.37198	0.14832
	-	301	Power law	1.29137	0.07376
	-	356	Power law	1.65713	0.03778
Encapsulated between hBN flakes	95 nm	98	Power law	1.34136	0.03019
	105 nm	163	Power law	1.25004	0.02185
	130 nm	153	Power law	2.11888	0.03959
	150 nm	60	Power law	1.73246	0.06282
	240 nm	29	-	-	-

1.6. FDTD simulations

The numerical simulation was calculated using the 3D finite difference time domain (FDTD) method. The refractive indices for glass and hBN are 1.4 and 2.1, respectively. The simulation wavelength is 600 nm-700 nm.

The simulation includes three factors: excitation intensity I, quantum efficiency ϕ and collection efficiency κ, as shown in Figure S5. Excitation intensity is referred to as the electric field intensity at the hBN/glass interface. The quantum efficiency is defined as the enhancement factor of the total dipole emission power of the emitter encapsulated by hBN compared to that in vacuum. The quantity was obtained using a 3D flux monitor to calculate the total emitted power, with a dipole source representing the emitter. The collection efficiency is defined as the fraction of the total dipole emission power collected by the objective, which was simulated using a 2D flux monitor to represent the realistic collection range of the objective. In the weak excitation regime, the total enhancement factor η is given by

$$\eta = I * \phi * \kappa,$$

For the excitation enhancement, a plane wave was used as the excitation source, with perfectly matched layer (PML) boundary conditions applied along the z-direction and periodic boundary conditions in the x- and y-directions. For quantum efficiency and collection efficiency enhancement, a dipole is employed as the source, with PML boundary condition in all xyz directions. The polarization of the dipole is in-plane oriented.[6,7]

The same location of high and low intensity regions was also obtained by calculating the thin-film interference conditions and considering the system as the Fabry-Pérot cavity with the fluorophore as the point source located in between the hBN and glass surfaces. Here, wave interference is due to the reflection and transmission from the glass and hBN surfaces. The following equation for constructive interference is used:

$$\Delta L = 2n_{hBN}\,d,$$

Where ΔL is the extra path travelled by the reflective wave, d is the hBN thickness and n_{hBN} is the refractive index of hBN. The phase difference between the direct and reflected wave is:

$$\Delta\phi(d) = \frac{4\pi n_{hBN}\, d}{\lambda}$$

Where λ is the emission wavelength of the fluorophore. From there the total intensity can be calculated as follows:

$$I(d) = \mid E_{total} \mid^2 = E_0{}^2 \mid 1 + re^{i\Delta\phi} \mid^2 = I_0 \cdot [1 + r^2 + 2rcos(\frac{4\pi n_{hBN}\, d}{\lambda})]$$

Where $I_0 = E_0{}^2$ is the intensity of the direct wave and r is the amplitude reflection coefficient from the hBN surface. In this formula only the first reflected and transmitted wave were taken into account. For more precision all other reflection and transmission terms also can be used in the simulation.

2. Supporting figures

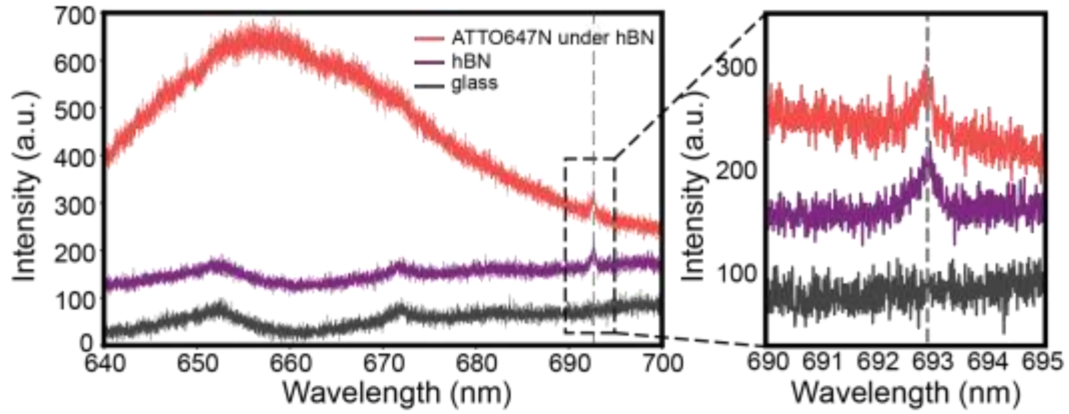

Figure S1. PL of ATTO647N under hBN and Raman peak of hBN on glass. (a) Full measured range. **(b)** Zoom-in into the hBN Raman peak.

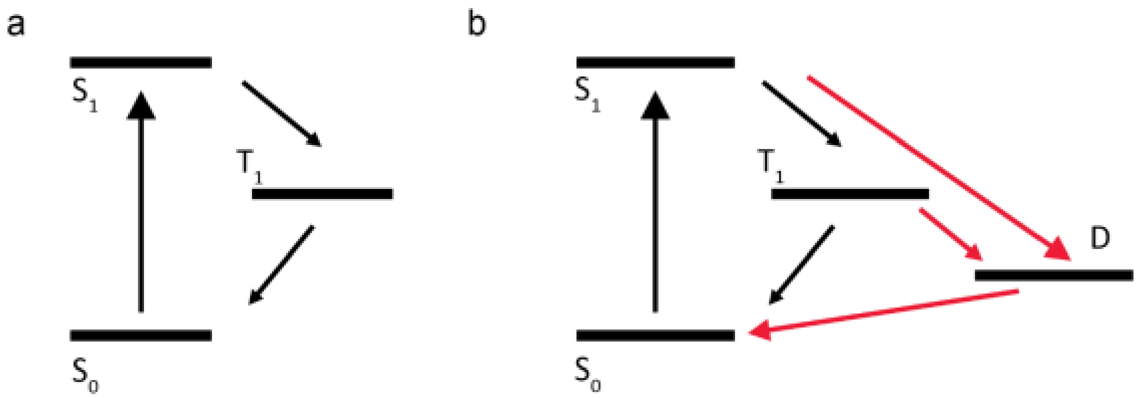

Figure S2. Jablonski diagram describing the formation of dark states for the fluorophore on different substrates and media. (a) Schematic Jablonski diagram for the fluorophore immobilized on the surface in buffer with photostabilizers. S_0 is the ground state, S_1 is the excited state. T_1 is the triplet state (dark state) – the only source of blinking. (b) Jablonski diagram for the fluorophore immobilized on glass or on hBN. D is the new dark (radical) state caused by the presence of the non-fluorescent traps. Red errors determine the pathways of D formation.

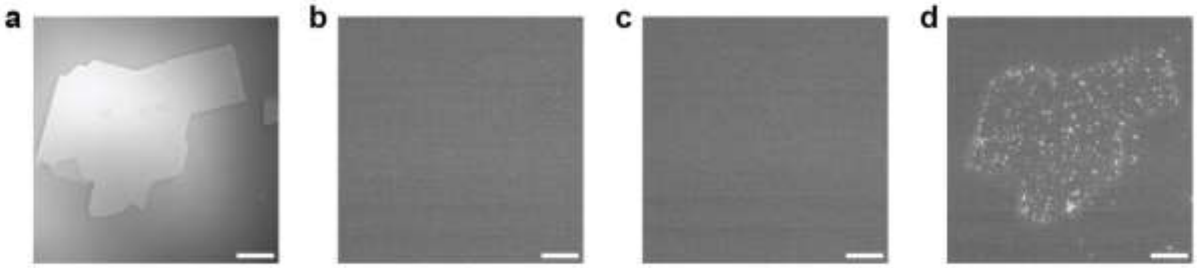

Figure S3. hBN background fluorescence and ATTO647N signal on top of hBN flake. (a) White light image of an hBN flake. **(b)** hBN flake in air excited by 640 nm laser. **(c)** hBN flake in buffer excited by 640 nm laser. **(d)** hBN flake with 5 pM ssDNA-ATTO647N in buffer. Scale bar is 10 μm.

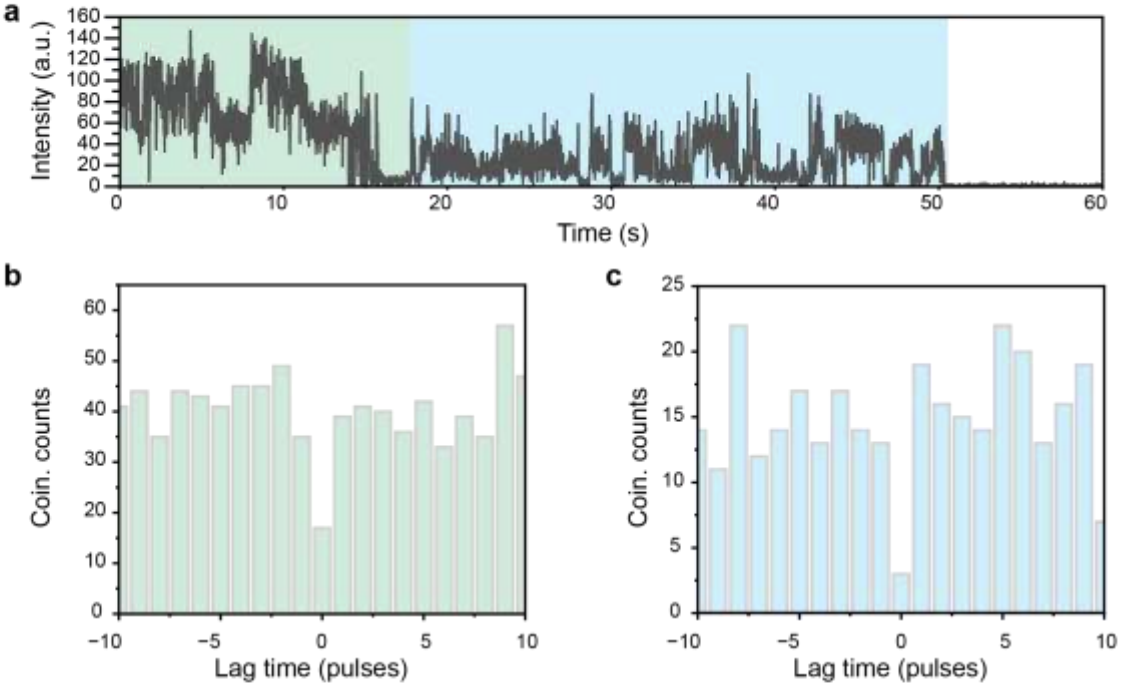

Figure S4. Second-order correlation function for multiple emitters. (a) Exemplary trace of the spot containing multiple molecules where green and blue colours indicate the parts with different intensity (so called, bleaching steps). **(b)** Coincident counts for the green region of the trace, showing clear multiple emitters correlation function. **(c)** Coincident counts for the blue region of the trace, showing clear single emitter correlation function.

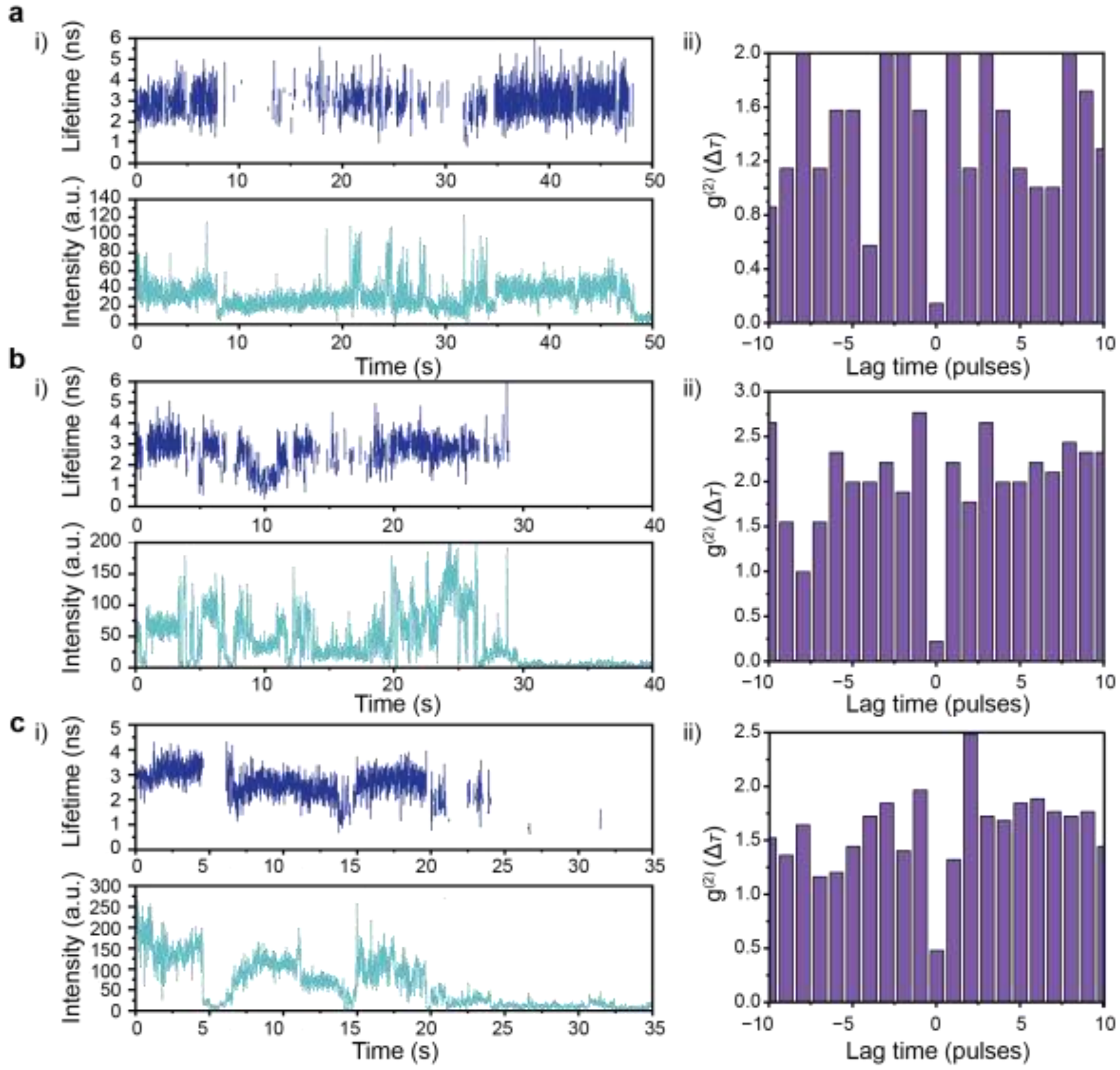

Figure S5. Dynamic quenching of ATTO647N. Lifetime and intensity change over time of ATTO647N deposited on top of hBN in the case of dynamic quenching for 3 different spots.

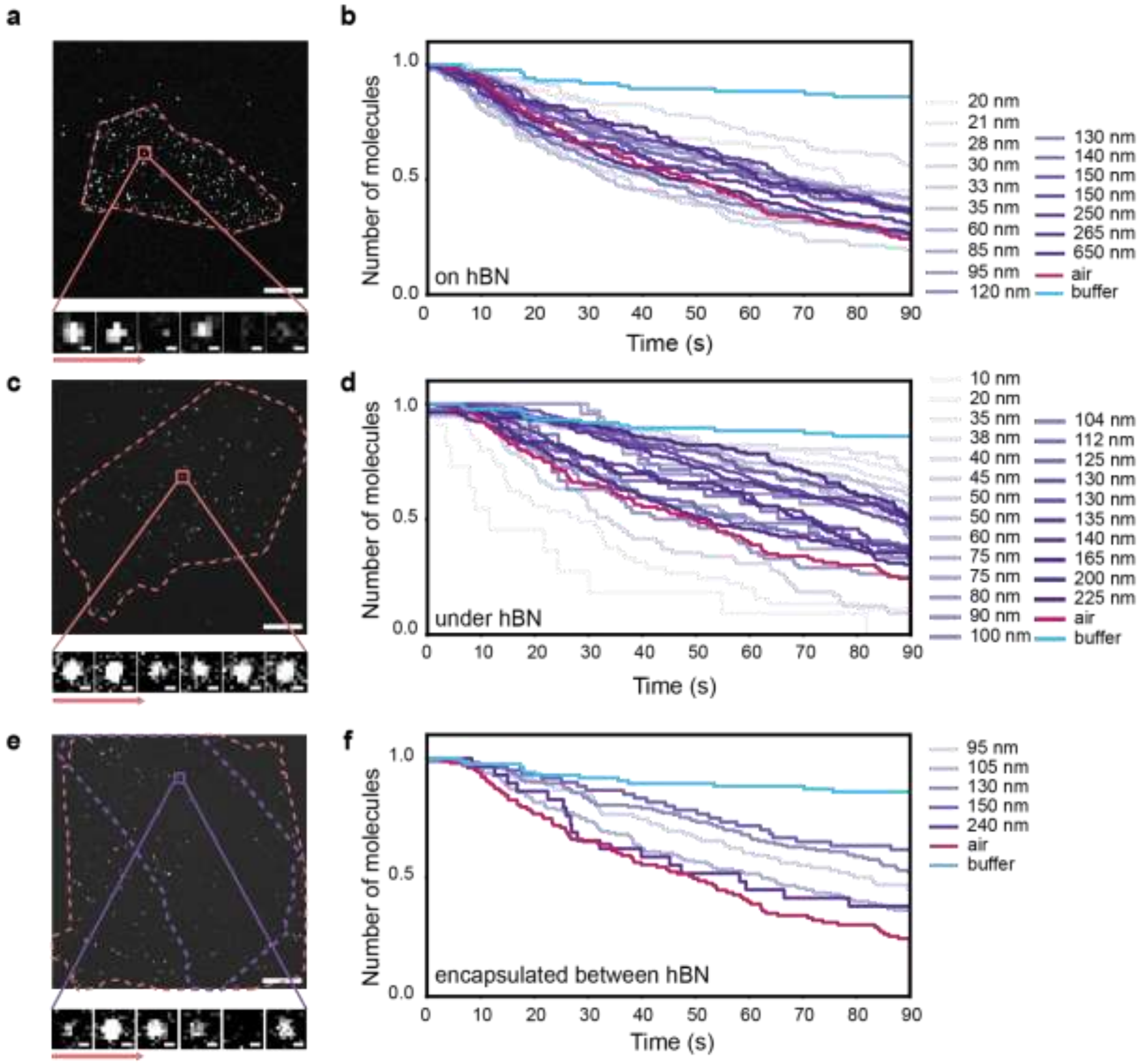

Figure S6. Normalized number of molecules as a function of time on and under hBN of different thickness. (a) hBN flake with fluorophore on top. Insets: individual fluorophore brightness change over time. **(b)** Normalized photobleaching curves as a function of hBN thickness for ATTO647N on hBN. Photobleaching curves in air (red) and in buffer (blue) are used as a reference. **(c)** hBN flake with fluorophores underneath. Insets: individual fluorophore brightness change over time. **(d)** Normalized photobleaching curves as a function of hBN thickness for ATTO647N under hBN. Photobleaching curves in air (red) and in buffer (blue) are used as a reference. **(e)** hBN flakes with encapsulated fluorophores. **(f)** Normalized photobleaching curves as a function of hBN thickness for ATTO647N encapsulated between different hBN flakes. Photobleaching curves in air (red) and in buffer (blue) are used as a reference.

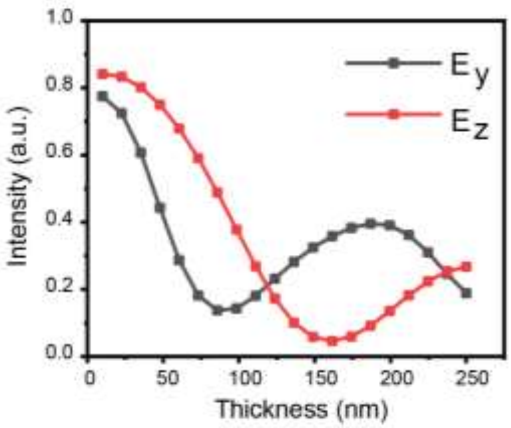

Figure S7. Comparison of fluorophore intensity with E_y and E_z polarization, simulated for fluorophores underneath the hBN surface.

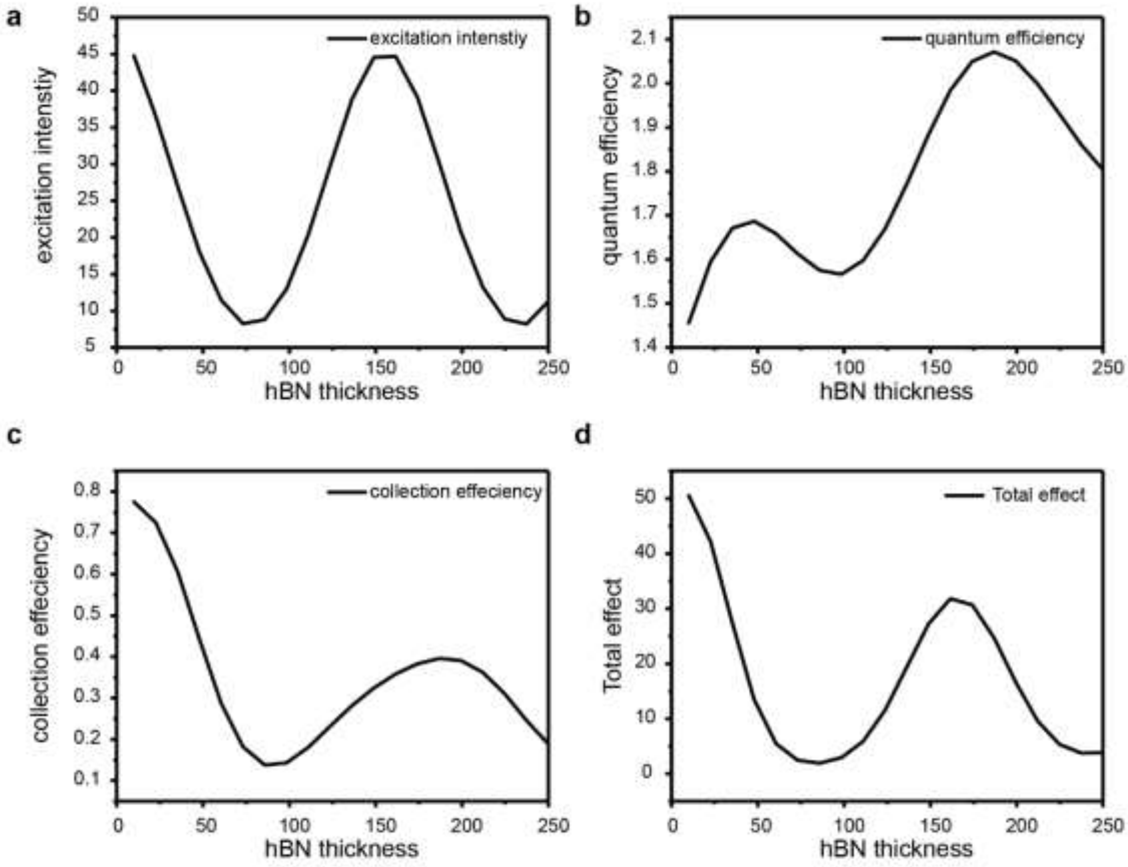

Figure S8. The effect of the excitation enhancement, quantum efficiency enhancement and collection efficiency enhancement on the fluorophore brightness under hBN. (a) excitation intensity change as a function of hBN thickness. **(b)** quantum efficiency change depending on hBN

thickness. **(c)** collection efficiency change depending on hBN thickness. **(d)** total intensity change as a function of on hBN thickness.

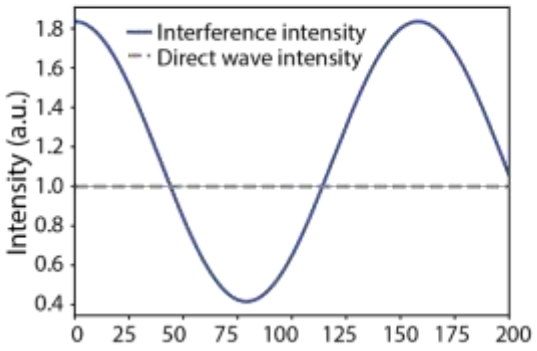

Figure S9. Thin-film interference in the system with hBN serving as Fabry-Pérot cavity. Intensity of the direct wave was set to 1 and is shown as the dashed line on the plot. Interference intensity calculated with the thin-film interference formulas is shown as the blue line.

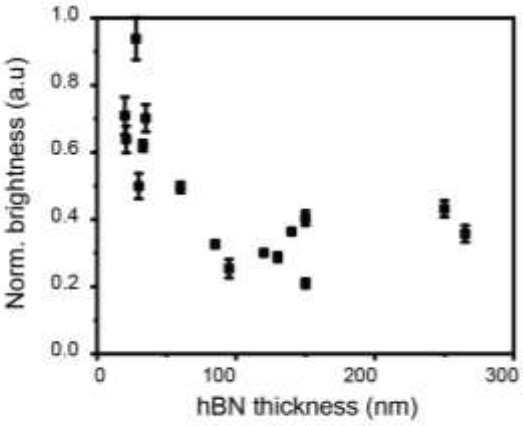

Figure S10. Brightness modulation of ATTO647N measured on top of hBN in air.

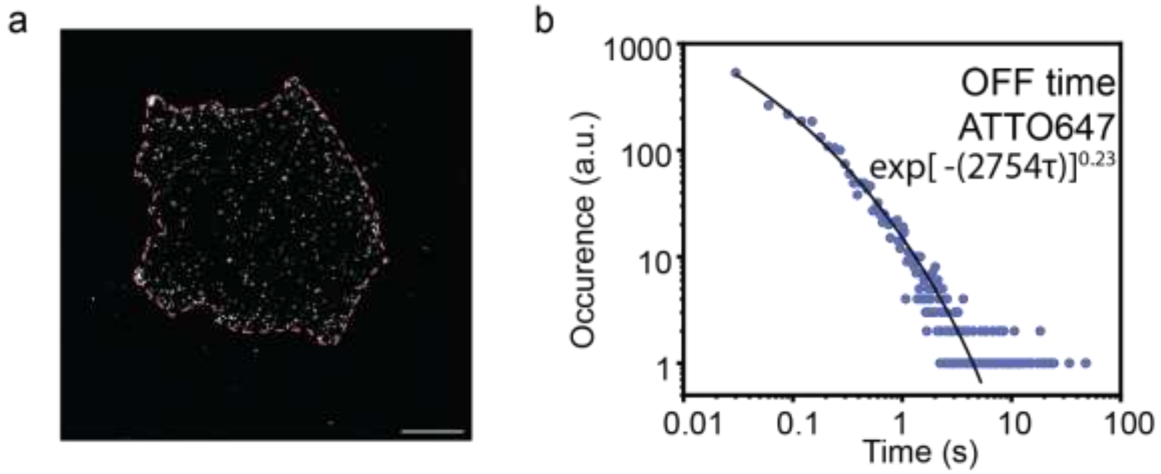

Figure S11. ATTO647 OFF time distribution. **(a)** Wide-field image of the hBN flake with the ATTO647-ssDNA molecules on top. Scale bar 10 µm. **(b)** OFF times distributions for ATTO647 fitted with a stretched exponential decay.

References

(1) Schell, A. W.; Tran, T. T.; Takashima, H.; Takeuchi, S.; Aharonovich, I. Non-linear excitation of quantum emitters in hexagonal boron nitride multiplayers. *Apl Photonics* **2016**, *1* (9). DOI: Artn 09130210.1063/1.4961684.

(2) Kumar, A.; Cholsuk, C.; Zand, A.; Mishuk, M. N.; Matthes, T.; Eilenberger, F.; Suwanna, S.; Vogl, T. Localized creation of yellow single photon emitting carbon complexes in hexagonal boron nitride. *Apl Mater* **2023**, *11* (7). DOI: Artn 07110810.1063/5.0147560.

(3) Smit, R.; Tebyani, A.; Hameury, J.; van der Molen, S. J.; Orrit, M. Sharp zero-phonon lines of single organic molecules on a hexagonal boron-nitride surface. *Nature Communications* **2023**, *14* (1). DOI: ARTN 796010.1038/s41467-023-42865-4.

(4) Schroder, T.; Bohlen, J.; Ochmann, S. E.; Schuler, P.; Krause, S.; Lamb, D. C.; Tinnefeld, P. Shrinking gate fluorescence correlation spectroscopy yields equilibrium constants and separates photophysics from structural dynamics. *Proc Natl Acad Sci U S A* **2023**, *120* (4), e2211896120. DOI: 10.1073/pnas.2211896120.

(5) Preus, S.; Noer, S. L.; Hildebrandt, L. L.; Gudnason, D.; Birkedal, V. iSMS: single-molecule FRET microscopy software. *Nat Methods* **2015**, *12* (7), 593-594. DOI: 10.1038/nmeth.3435.

(6) Wenger, J. Fluorescence Enhancement Factors on Optical Antennas: Enlarging the Experimental Values without Changing the Antenna Design. *Int J Opt* **2012**, *2012*. DOI: Artn 82812110.1155/2012/828121.

(7) Jameson, D. M.; Ross, J. A. Fluorescence Polarization/Anisotropy in Diagnostics and Imaging. *Chem Rev* **2010**, *110* (5), 2685-2708. DOI: 10.1021/cr900267p.
